# Influence of hemodialysis on pramipexole pharmacokinetics: Lessons from two cases and literature review 

**DOI:** 10.5414/CNCS109641

**Published:** 2019-03-22

**Authors:** Nicolas Hanset, Philippe Hantson, Franck Saint-Marcoux, Arnaud Devresse, Michel Jadoul, Laura Labriola

**Affiliations:** 1 Department of Nephrology, Cliniques Universitaires Saint-Luc, Université Catholique de Louvain Brussels,; 2 Department of Intensive Care, Cliniques Universitaires Saint-Luc,; 3 Louvain Center for Toxicology and Applied Pharmacology, Université Catholique de Louvain, Brussels, Belgium, and; 4 Department of Pharmacology and Toxicology, CHU Limoges, Limoges, France

**Keywords:** pramipexole, hemodialysis, pharmacokinetics

## Abstract

Background: Restless legs syndrome (RLS) is not a rare condition in patients on long-term dialysis. Pramipexole is a small molecule used in the treatment of idiopathic and uremic RLS. Although some information concerning the efficacy and safety of pramipexole in uremic patients is available, data concerning the pharmacokinetics of pramipexole in hemodialysis (HD) are lacking. Following the occurrence of accidental pramipexole intoxication in a chronic HD patient, we were concerned about the efficacy of HD in removing pramipexole. Our aim was thus to assess plasma pramipexole concentrations and pramipexole clearance in a stable chronic HD patient without any residual kidney function. Materials and methods: Our patient was a 63-year-old man on chronic HD for 5 years who had been treated uneventfully with oral pramipexole for uremic RLS since then. During a routine 4-hour high-flux HD session, blood, ultrafiltrate, and dialysate samples were collected every hour to determine pramipexole concentrations over time. Results: Pramipexole blood concentrations ranged from 12.1 to 23.9 µg/L. Pramipexole reduction ratio was 32.5%. Mean dialytic clearance of pramipexole was 76.8 mL/min. Postdialysis rebound was 5.6%. Conclusion: In the absence of any side effect, pramipexole blood concentrations at steady state were 2- to 4-fold higher than those observed in subjects with normal kidney function. Like other drugs with a high volume of distribution, pramipexole was poorly removed by HD. Therefore, HD is not recommended as a treatment option for pramipexole intoxication in patients with a glomerular filtration rate superior to 30 mL/min/1.73m².

## Introduction 

Restless legs syndrome (RLS) is not a rare condition in patients on long-term dialysis. Reported prevalence in hemodialysis (HD) is variable (6.6 – 49%), due to differences in diagnostic methods [1]. Pramipexole, an oral non-ergoline dopamine agonist, has selective activity for the dopamine D3 receptor and is used as an antiparkinsonian agent. It has been approved in 2006 in the EU [2] and the US [3] for the treatment of moderate to severe idiopathic RLS in adults [4, 5]. The metabolism of pramipexole is minimal and the drug is excreted virtually unchanged in the urine by tubular secretion [6]. Although some information concerning the efficacy and safety of pramipexole in uremic patients is available [7], data concerning the removal of pramipexole during HD are lacking, and recommendations concerning its use in HD patients are extrapolated from data obtained in the general population. 

We previously reported the case of a 79-year-old anuric chronic HD patient, who accidentally suffered a pramipexole overdose (the dosage was increased from 0.09 to 1.4 mg/d and administered 2 days in a row) and presented an acute deterioration of the level of consciousness with hypercapnic respiratory failure, necessitating admission to intensive care unit and non-invasive ventilation [8]. Pramipexole intoxication was suspected as a potential etiology. The medication was thus withdrawn, and daily intermittent HD was performed. The patient’s neurological and respiratory status returned to normal very slowly from the third day. Maximal pramipexole blood concentration was 7.8 µg/L (measured 51 hours after the last dose), and clearance by HD was measured at 73.3 mL/min. 

The pharmacokinetics of drugs vary widely in HD, depending on characteristics, such as molecular weight, protein binding capacity, and volume of distribution. Thus, one specific molecule may tend to accumulate or, on the contrary, be excreted by HD, which can result in a major impact on safety and therapeutic efficacy, respectively. Even though one might assume dialyzability and pharmacokinetic profiles on the basis of the key features mentioned above, confirmative data are needed for a safe use of drugs in HD patients. In this setting, we were concerned about the efficacy of HD in clearing pramipexole. Given its small molecular weight and a low protein binding [6], significant HD removal could be expected. However, the efficacy of HD in pramipexole clearance is likely to be mitigated by the huge volume of distribution of pramipexole (7 L/kg) [9]. Our purpose was thus to assess plasma pramipexole concentrations and pramipexole clearance and removal in a chronic HD patient without residual kidney function. 

## Materials and methods 

### Patient and hemodialysis 

Our patient was a 63-year-old Caucasian man on in-center chronic HD for 5 years for end-stage renal disease secondary to autosomal dominant polycystic kidney disease. Relevant medical history included combined kidney and liver transplantation 5 years earlier, hypertension, post-transplant lymphoproliferative disease, and uremic RLS diagnosed 5 years earlier according to international RLS diagnostic criteria [10]. He weighed 80.3 kg at a height of 178 cm. He had been treated with oral pramipexole 0.18 mg twice daily for the last 5 years, resulting in a complete remission of RLS, without any side effect. He was dialyzed 4 hours 3 times a week. His vascular access was a native upper arm arteriovenous fistula (AVF) created 5 years earlier and cannulated using the buttonhole technique with blunt needles. Access blood flow was measured at 3,065 mL/min using saline dilution (Transonic Systems Inc., Ithaca, NY, USA), without recirculation. 

All the measurements for this pharmacokinetic study were performed during the mid-week HD session using a GENIUS 90 Therapy System (Fresenius Medical Care, Bad Homburg, Germany) with a HDF 600 hollow-fiber high-flux polysulfone dialyser (Fresenius Medical Care) for 4 hours. Dialysate composition was: sodium 138 mMol/L, potassium 4 mMol/L, calcium 1.5 mMol/L, bicarbonate 32 mMol/L, acetate 3.0 mMol/L, magnesium 0.5 mMol/L, and glucose 100 mg/dL. Blood flow and dialysate flow rates were set at 340 mL/min. Dialysate temperature was 36 °C. Anticoagulation was achieved using nadroparin calcium 0.6 mL. The dialyzer was not reused. 

### Sample handling and analysis 

The patient received the last pramipexole dose 4 hours before starting the HD session. Blood samples for pramipexole measurements were drawn from the blunt needle placed at the arterial puncture site of the AVF 2 (H_–2_) and 1 (H_–1_) hour(s), and immediately before (H_0_), the HD session. During HD, blood samples were collected hourly (H_1_, H_2_, H_3_) from the arterial needle. Blood was also taken from the venous needle at H_1_ and H_3_. Post-HD samples were obtained from the arterial needle tubing after disconnection (H_4_), as well as 30 and 120 minutes after the end of the HD session. Ultrafiltrate was collected every hour from the ultrafiltrate vessel, after stirring its content. Spent dialysate was collected hourly from the efferent dialysate port and sampled immediately after dialysis. All samples described above were collected in heparinized blood collection tubes, then centrifuged and stored in a –20 °C freezer until assayed (Department of Pharmacology and Toxicology, CHU Limoges, Limoges, France). 

### Analytical methods 

Concentrations of pramipexole were determined by using a liquid chromatography coupled to tandem mass spectrometry method (LC-MS/MS). The chromatographic system consisted of two Shimadzu LC-30 AD pumps (Nexera X2), a CTO 20AC oven, and a SIL-30 AC-MP autosampler (Shimadzu, Marne-la-Vallée, France). Chromatographic separation was performed using a Pinnacle DB PFPP, 1.9 μm (50 × 2.1 mm I.D.) column (Restek, Lisses, France). A Shimadzu 8050 triple quadrupole mass spectrometer (Marne-la-Vallée, France) was used in the positive electrospray ionization mode, and multiple-reaction monitoring (MRM) transitions were as follows: 212.10-153.05; 212.10-111.00; 212.10-126.05. The laboratory of Pharmacology-Toxicology of the Limoges University Hospital works in accordance with the International Standards Organization (ISO) 15189 standard (accreditation number: 8-2607), and the method was developed according to an accredited validation protocol. Among the usual acceptance criteria, Coefficient of variability (CV) values were less than 15% in the range 2.5 – 500 µg/L for intra-assay and the inter-assay precision and accuracy. Creatinine measurements were performed on a Cobas 8000 analyzer module c702 (Roche Diagnostics, Basel, Switzerland), with the Jaffe generation 2 method, a kinetic colorimetric assay, according to the manufacturer recommendations. 

### Pharmacokinetic analysis 

Plasma pramipexole reduction ratio (PRR) was calculated according to the following formula: 


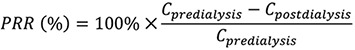


where C_predialysis_ and C_postdialysis_ are blood concentrations of pramipexole at the start and the end of HD session, respectively. 

The total mass of pramipexole removed by HD (XH_p_) was estimated by the following equation: 


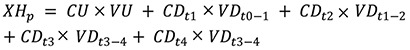


where CU is the pramipexole concentration in ultrafiltrate, VU is the total volume of ultrafiltrate, CD is the pramipexole concentration in the efferent dialysate, VD is the volume of dialysate, and t is the time interval at which measures were made. 

On the basis of the two previous equations, total pramipexole blood mass before HD (XB_p_) was calculated as: 


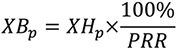


Dialytic clearance of pramipexole (Cl_dial_) was derived from the Fick principle, using the following equation: 


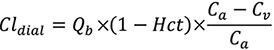


where Q_b_ is blood flow rate, Hct is hematocrit, C_a_ is the predialyzer blood pramipexole concentration, and C_v_ is the postdialyzer blood pramipexole concentration. 

Rebound (Rbd) was calculated using the following equation: 


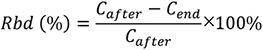


where C_end_ is the blood pramipexole concentration measured on the predialyzer (arterial) port at the end of dialysis and C_after_ is the maximal pramipexole blood concentration measured after dialysis by direct venipuncture. 

## Results 

**Figure 1.**
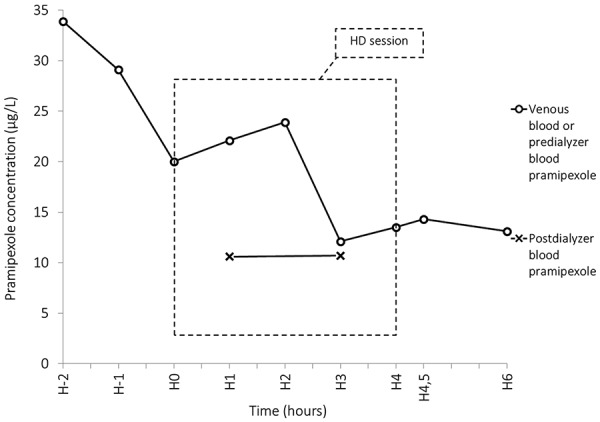
Effect of HD on pramipexole concentrations.

Characteristics of the HD session are depicted in Table 1. Pramipexole and creatinine concentrations over time are reported in Table 2 and Figure 1. 

Pramipexole blood concentrations ranged from 12.1 to 23.9 µg/L. As shown in Figure 1, the drug concentrations, measured starting 2 hours after oral intake, were not stable prior to the HD session, a finding suggestive of ongoing absorption and distribution of the drug. Pramipexole reduction ratio was 32.5%. Total mass of pramipexole removed by HD was estimated at 469 µg. Total blood mass of pramipexole before HD was estimated at 1,466 µg. Mean dialytic clearance of pramipexole was 76.8 mL/min. Postdialysis rebound was 5.6%. 

## Discussion 

Pramipexole is a synthetic aminobenzothiole derivative with selective agonist action on D_2_ – D_3_ presynaptic dopamine autoreceptors and thus beneficial effects on parkinsonian symptoms [11]. Although its mechanism of action in restless legs syndrome remains unclear [4], pramipexole has been found to be effective as symptomatic treatment of idiopathic and uremic RLS [5, 7]. 

Pramipexole is rapidly absorbed, has an oral bioavailability > 90%, and undergoes minimal metabolism [6]. It has a linear pharmacokinetic, with maximal plasma concentration achieved in 1 hour [9]. In individuals with normal kidney function, serum half-life is 8 – 14 hours, whereas in end-stage renal disease, half-life extends up to 38 hours [12]. 

In subjects with normal renal function, ~ 90% of a dose of pramipexole is excreted via renal tubular secretion, unchanged into the urine [4, 9]. As renal function decreases, pramipexole clearance correlates with creatinine clearance (411 ± 85.9 mL/min, 297 ± 57.2 mL/min, 192 ± 52.5 mL/min and 131 ± 22.2 mL/min in subjects with normal, mild (creatinine clearance 66.0 ± 8.9 mL/min), moderate (creatinine clearance 42.3 ± 6.8 mL/min) and severe (creatinine clearance 21.9 ± 3.9 mL/min) renal impairment, respectively) [12]. Given its small molecular weight (302.3 Da) and a low protein binding (< 20%) [6], significant HD removal could be expected. However, HD efficacy seems to be very low, probably due to the huge volume of distribution of pramipexole (7 L/kg) [9], which indicates that a significant amount of the drug is distributed in the extravascular space, i.e., not directly accessible for removal by HD. 

In the only report focusing on that subject, including three chronic HD patients treated with pramipexole 0.250 mg twice daily, Wright et al. document that less than 9% of the dose was cleared by a 3-hour dialysis. Pramipexole clearance was not measured [12]. Dexpramipexole, also known as R-(+)-Pramipexole, is an enantiomer of pramipexole recently developed, and used as neuroprotective agent in amyotrophic lateral sclerosis, with pharmacokinetic features similar to those of pramipexole. In a study evaluating the impact of renal failure and HD on dexpramipexole concentration, high-flux HD decreased dexpramipexole plasma concentration by 25% [13]. In the present case, and in our other previously reported patient [8], pramipexole reduction ratios by HD were 32.5 and 44.3%, respectively, and mean dialytic clearances of pramipexole were estimated at 76.8 and 73.3 mL/min, respectively. Postdialysis rebound was low (~ 5%), probably owing to an early start of the redistribution, before the end of dialysis, because of high volume of distribution and continuous high refilling rate. Even though concentrations were measured during the distribution phase of the drug, the simultaneous measurement of pramipexole concentrations in post-dialyzer blood, ultrafiltrate and dialysate provides unquestionable information about the effects of HD on drug removal. In the light of those findings, given the poor efficacy of HD in pramipexole removal, HD is not recommended as a treatment option in intoxicated patients with an estimated glomerular filtration rate superior to 30 mL/min/1.73m². 

Data on pramipexole blood levels are scanty, especially regarding RLS. In healthy volunteers taking 0.375 – 4.5 mg/d, mean trough plasma concentrations ranging around 1.5 – 5.0 µg/L have been measured, with a peak plasma concentration of ~ 6.0 µg/L [9]. Although uncommon, adverse events such as nausea, dizziness, headache, sleep disorders (insomnia, somnolence, sudden onset of sleep), and psychiatric reactions (mainly hallucinations) might occur, mainly in association with dose escalation [9, 11]. There is limited information on pramipexole overdose [14, 15], but, since tolerance appears rapidly, slow titration is indicated [9]. In HD patients with uremic RLS, treatment with lower doses of pramipexole (0.125 – 0.5 mg/d) was effective, with no major adverse event [7]. In our patient, under long-established daily dose of 0.36 mg, plasma concentrations of pramipexole ranged from 12.1 to 23.9 µg/L, 2- to 4-fold higher than those reported in healthy volunteers in the absence of apparent toxicity. Total blood pramipexole mass before HD was estimated to be equivalent to approximately four daily doses. These findings suggest a propensity of pramipexole to accumulate despite HD, in association with a strong tolerance phenomenon, and question the reliability of serum concentrations measurement to assess intoxication in HD patients. Even if based on low-level evidence, we agree with the experts’ recommendations to use doses ranging from 0.125 to 1.6 mg/d in patients with no significant renal impairment, to initiate treatment at low doses with slow titration until symptoms relief is obtained, and, in dialysis patients, to consider 0.75 mg as a maximal daily dose [16]. 

## Conclusion 

Pharmacokinetic features of pramipexole in HD, assessed by blood and dialysate measures, correlate with those expected from a theoretical point of view. Surprisingly though, despite the lack of any apparent side effect, pramipexole blood concentrations at steady state exceeded by far those observed in subjects with normal kidney function. This suggests a propensity to drug accumulation and tolerance along with long-term administration and questions the reliability of plasma pramipexole concentration measurement to diagnose intoxication. We confirm a poor efficacy of HD in pramipexole clearance (close to 80 mL/min), which does not outperform the renal pramipexole clearance observed in patients with moderate chronic kidney disease. Therefore, HD is not recommended as a treatment option for pramipexole intoxication in patients with an estimated glomerular filtration rate superior to 30 mL/min/1.73m². 

## Compliance with ethical standards 

All patients cited in this paper gave written informed consent to participate in the study. 

## Funding 

None of the authors received any support/funding for this study. 

## Conflict of interest 

Prof. L. Labriola reports personal fees (lecture fees) from Fresenius, personal fees (lecture fees) from Amgen, personal fees (travel support) from Shire, personal fees (travel support) from Bellco, personal fees travel support) from Janssen-Cilag, outside the submitted work. Prof. M. Jadoul reports consulting fees from Amgen, consulting fees from Astellas, consulting fees from Fresenius, consulting fees from GSK, consulting fees from MSD, consulting fees from Sanofi, traveling support from Amgen, grants from Alexion, grants from Amgen, grants from Baxter, grants from Otsuka, grants from Janssen-Cilag, grants from Roche, lecture fees from Abbvie, lecture fees from Menarini, lecture fees from MSD, lecture fees from Amgen, outside the submitted work. Dr. N. Hanset, Prof. P. Hantson, Prof. F Saint-Marcoux, Dr. A. Devresse: no conflict of interest to declare. 

**Table 1.** Hemodialysis session characteristics.

Actual hemodialysis time (min)	240
Blood flow rate (mL/min)	340
Dialysate flow rate (mL/min)	340
Ultrafiltration rate (mL/h)	320
Actual total ultrafiltration volume (mL)	1,170
Creatinine reduction ratio (%)	64.9%

**Table 2.** Pramipexole and creatinine concentrations over time.

Time (hours)	Predialysis		Peridialysis					Postdialysis	
	H_–2_	H_–1_	H_0_	H_1_	H_2_	H_3_	H_4_	H_4.5_	H_6_
Pramipexole (µg/L)									
Predialyzer blood	33.9	29.1	20.0	22.1	23.9	12.1	13.5	14.3	13.1
Postdialyzer blood				10.6		10.7			
Dialysate				10.9	5.1	3.6	5.1		
Ultrafiltrate				11.4	6.8	5.2	4.5		
Creatinine in predialyzer blood (mg/dL)									
			9.51				3.34		

H = hour.
